# Viral hepatitis detection through emergency hospital departments in a Spanish region with high immigration: implications for elimination strategies

**DOI:** 10.1186/s40249-026-01472-3

**Published:** 2026-07-06

**Authors:** Carme López, Maria Àngels Gispert, Emma Picart, Rosa Durández, Dúnia Pérez del Campo, Miguel Torralba-Calero, Francesc Xavier Queralt, Robert Carreras-Torres, Javier Pamplona

**Affiliations:** 1 Department of Gastroenterology, Josep Trueta University Hospital (Hospital Universitari de Girona Dr Josep Trueta, Institut Català de La Salut (ICS)), Girona, Spain; 2 Department of Emergency Services, Josep Trueta University Hospital (Hospital Universitari de Girona Dr Josep Trueta, Institut Català de La Salut (ICS)), Girona, Spain; 3 Department of Emergency Services, Santa Caterina Hospital (Hospital Santa Caterina de Salt, Institut d’Assistència Sanitària (IAS)), Girona, Spain; 4 Department of Gastroenterology, Santa Caterina Hospital (Hospital Santa Caterina de Salt, Institut d’Assistència Sanitària (IAS)), Girona, Spain; 5 Territorial Clinical Laboratory of Girona (Laboratori Clínic Territorial de Girona (LCTG), Institut Català de La Salut (ICS)), Girona, Spain; 6 Digestive Diseases Group, Girona Biomedical Research Institute (Institut d’Investigació Biomèdica de Girona Dr Josep Trueta (IDIBGI-CERCA)), Girona, Spain

**Keywords:** Hepatitis B virus, Hepatitis C virus, Emergency department screening, Migrant populations

## Abstract

**Background:**

Chronic infection with hepatitis B virus (HBV) and hepatitis C virus (HCV) remains a major global public health problem, as many infected individuals remain undiagnosed. Emergency departments (ED) represent a strategic setting for opportunistic screening. This study aimed to evaluate implementation strategies of opportunistic screening in ED within the Girona Health Region in Spain.

**Methods:**

A prospective observational study was conducted in the ED of the Hospital Universitari de Girona Dr. Josep Trueta (HUGJT) and the Hospital Santa Caterina (HSC) between October 2023 and January 2024. Adults older than 18 years who required blood testing for clinical reasons were screened. In HUGJT screening was automated within the electronic system (opt-out strategy), while in HSC it was performed following a specific request from the attending physician. Comparisons of screening outcomes between hospitals and patient characteristics were evaluated using non-parametric tests.

**Results:**

A total of 1695 patients from HUGJT (54.3% coverage) and 303 patients from HSC (6.9% coverage) with valid screening results were included. In HUGJT, antibodies to HCV were detected in 37 individuals (2.2%); while active infection was confirmed in 9 patients (0.5%) (50.0% were previously undiagnosed), being more frequent among individuals with a history of heroin (20.0%). Hepatitis B surface antigen was detected in 11 patients (0.6%) (60.0% had no prior diagnosis), being more common among individuals born outside Spain, particularly those from South-Saharan Africa (7.1%), North Africa (3.2%), and Eastern Europe (2.2%).

**Conclusions:**

Opt-out opportunistic screening in ED is effective for identifying undiagnosed infections and facilitating linkage to care.

**Graphical Abstract:**

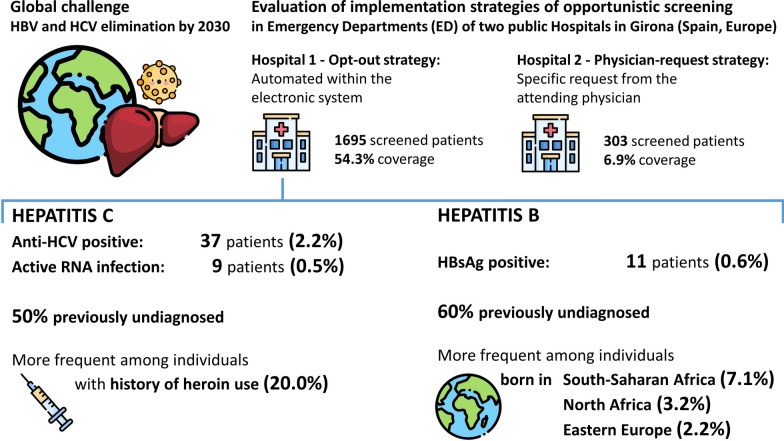

**Supplementary Information:**

The online version contains supplementary material available at 10.1186/s40249-026-01472-3.

## Background

Infection with hepatitis B virus (HBV) and hepatitis C virus (HCV) represents a major global public health challenge, with high morbidity and mortality linked to chronic liver disease and millions of affected individuals world-wide, many of whom remain undiagnosed [1, 2]. Early detection and effective linkage-to-care (LTC) are essential to achieving the elimination targets set by the World Health Organization (WHO) for 2030, which include reducing the number of new infections by 90% and the number of mortalities by 65% [3]. Within this global strategy, the “micro-elimination” approach focuses efforts on groups with higher infection prevalence, particularly vulnerable populations [4]. Early diagnosis and timely access to treatment in these groups are critical to accelerating viral hepatitis elimination and reducing the overall disease burden at global, national and regional levels [5, 6].

In Spain, most referral hospitals have the necessary resources to provide comprehensive one-step viral hepatitis testing from a single sample. This involves on-site clinical laboratories performing Hepatitis B surface antigen (HBsAg) testing and anti-HCV antibody testing using serum specimens. For hepatitis C, samples that test positive for anti-HCV antibodies are subsequently assessed for active infection by HCV RNA testing on the same serum sample. Emergency departments (EDs) are a strategic setting for HBV and HCV screening, as they often care for individuals who do not regularly engage with primary care services and who may have unrecognized risk factors [5, 7–10]. Within this context, epidemiological surveillance, vaccination, and early detection in EDs emerge as key components for reducing disease burden and ensuring timely referral to specialist care [11]. This approach is supported by existing evidence on the value of opportunistic screening in EDs and its contribution to viral elimination strategies. For instance, the estimated national prevalence, as multi-parameter evidence synthesis [12], provided by the Spanish Ministry of Health by February 2024 was 0.8% [95% confidence interval (*CI*): 0.6–1.1] for anti-HCV antibody testing, 0.1% (95% *CI*: 0.03–0.2) for active HCV-RNA positive testing, and 0.2% (95%* CI*: 0.1–0.3) for HBsAg positive testing [13]. However, recent studies performed in the ED of reference Hospitals in Barcelona and Almeria raised the estimate till 0.4–0.7% for active HCV and 0.5% for HBV [14, 15]. This strategy has been associated with greater coverage, improved efficiency in identifying active infections, and effective referral and continuity-of-care systems [14, 16–20].

The Health System of the Girona Health Region (Regió Sanitària de Girona—RSG), covering about 725,000 inhabitants, has prioritized screening programs targeting subgroups with higher prevalence, such as people who inject drugs (PWID) and prisoners [21]. However, the immigrant population, defined as individuals born outside Spain but residing in the country, has increasingly drawn attention as a group requiring further analysis regarding access barriers and opportunities for early diagnosis. This is particularly relevant despite Spain’s so-called universal healthcare system, which provides care regardless of legal residency status, transit situation, or the possibility of reimbursement from the individual’s country of origin in cases of temporary stay. For instance, HCV prevalence is higher (3.1%) in South-Saharan African immigrants living in Spain compared with other immigrant communities [22]. While immigrants represent the 16.1% of the general population in Catalonia (North-East region of Spain), the proportion raises to 20.5% and 38.5% in the RSG main city and metropolitan area, respectively [23]. In Spain, evidence suggests that migrant populations may exhibit different patterns of healthcare use and diagnosis compared with native populations, including lower utilization of primary and specialized care and a higher reliance on EDs, regardless of the immigrants’ age range or countries of origin. [24, 25]. This underscores the need to develop screening strategies adapted to the RSG regional context and to the care dynamics within EDs.

This study was conducted in the EDs of two public hospitals within the RSG: the Hospital Universitari de Girona Dr. Josep Trueta (HUGJT), located in the main city of the RSG, and the Hospital Santa Caterina (HSC), located in the RSG metropolitan area. In both hospitals, opportunistic screening for HBV and HCV was performed. However, at HUGJT, testing was automatically integrated into the electronic health record (EHR) system and carried out alongside routine blood tests, whereas at HSC, testing was requested at the clinician’s discretion without a predefined screening protocol. The aims of the study were: (i) to compare the outcomes of the two opportunistic screening strategies; (ii) to estimate the seroprevalence of active infection in the overall population and relevant subgroups; and (iii) to characterize associated risk profiles.

## Methods

### Study design

This prospective, observational study included a total of 2001 adults (> 18 years) attending the EDs, between October 2023 and January 2024, of the Hospital Universitari de Girona Dr. Josep Trueta (HUGJT) and the Hospital Santa Caterina (HSC), both in Girona (Spain). In both hospitals, inclusion criteria comprised requiring blood tests for another clinical indication and providing verbal consent, while exclusion criteria included being under 18 years of age or declining participation in the screening. At HUGJT, screening was automated within the EHR system. In contrast, at HSC, screening was performed following a specific request from the attending physician.

### Laboratory analysis

Screening was performed using HBsAg testing for HBV and anti-HCV antibody testing (Alinity-i HBsAg Qualitative II & Alinity-i anti‐HCV; Abbott Laboratories; USA). Blood test were performed in the Laboratori Clínic Territorial de Girona-Institut Català de la Salut (LCTG-ICS). Diagnostic confirmation of active infection in patients with a positive anti-HCV result was carried out using HCV RNA testing (cobas® HCV; Roche; Switzerland).

### Linkage to care and treatment

Positive results were communicated directly to the responsible clinical team, and referral pathways to specialized care were activated for newly diagnosed cases or those not previously linked to care, following clinical and community integration models from the European Centre for Disease Prevention and Control (ECDC) and international experience in urban settings [20, 26]. At the point of care, patients with a positive HBsAg result underwent confirmatory testing, including hepatitis B e antigen and core antibody assessment. Patients diagnosed with active infection received appropriate treatment, consisting of direct-acting antivirals (DAAs) for HCV infection or nucleos(t)ide analogues for HBV infection, as indicated by the European Association for the Study of the Liver (EASL) [27, 28].

### Data collection and storage

Screening results, referrals to specialist care, and follow-up outcomes were documented. Demographic, epidemiological, and clinically relevant variables were collected for the analysis of prevalence and characterization of risk profiles, including age, sex, country of origin, relevant medical history, substance use, and associated comorbidities. All data were stored in a RedCAP project of the research centre account (Insititut d’Investigació Biomèdica de Girona Dr Josep Trueta – IDIBGI), and managed in accordance with institutional confidentiality standards. This study was approved by the Clinical Research Ethics Committee on Medicinal Products (CEIM) of IDIBGI (Record 09/2023; Code 2022.175). Confidentiality and secure data handling were ensured throughout the study.

### Data analysis

Statistical analysis was performed using the software R 4.5.2 (R Core Team, Auckland, New Zealand). The distribution of continuous variables was expressed as median and interquartile range, and the proportion (%) of categories for categorical variables. Associations between positive anti-HCV and HBV markers and demographic and clinical variables were assessed using non-parametric tests: the Kruskal-Wallis test for continuous variables (age and body mass index) and the chi-square test for categorical variables. To account for multiple testing, the significance threshold for association was defined as *P* < 0.01, which were highlighted in bold.

## Results

### Patients recruitment

**Fig. 1:**
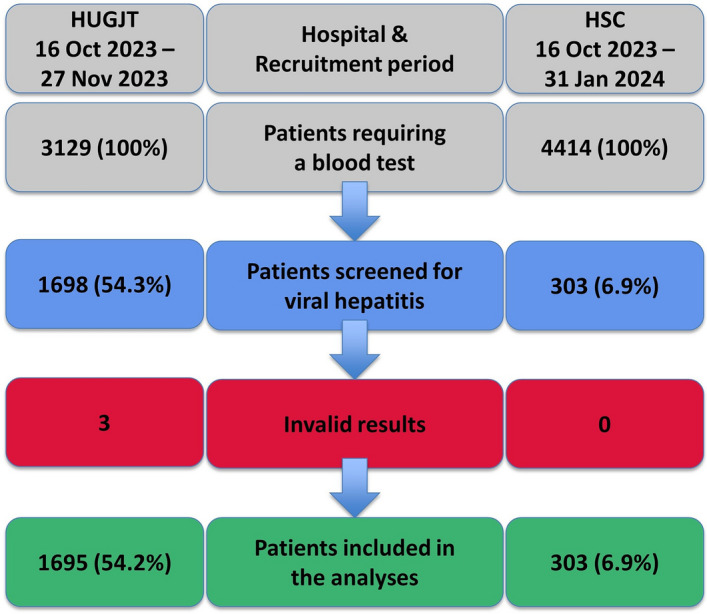
Flowchart of opportunistic screening in the Hospital Universitari de Girona Dr. Josep Trueta (HUGJT) and the Hospital Santa Caterina (HSC)

During the study period, 3129 individuals required blood analysis at EDs of HUGJT, of whom 1698 (54.3%) were screened for viral hepatitis. At EDs of HSC, 303 individuals (6.9%) out of 4414 with blood test were screened. Hepatitis testing valid results were obtained for 1998 screened patients. Figure 1 depicts the flowchart of the screening periods, including the number of patients requiring blood tests, those screened, and the valid results obtained.

### Demographic and clinical parameters

**Fig. 2:**
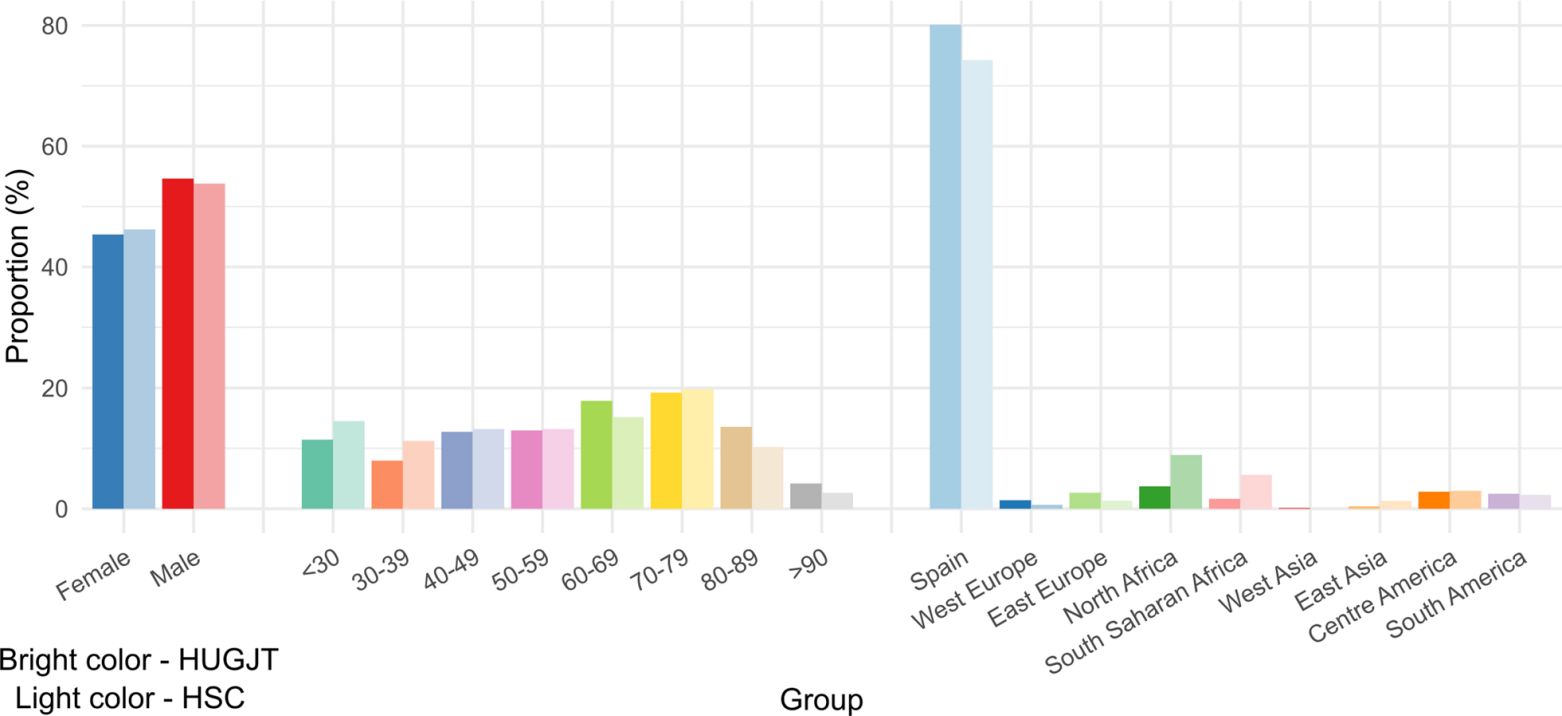
Demographic parameters across the two hospitals. HUGJT: Hospital Universitari de Girona Dr. Josep Trueta; HSC: Hospital Santa Caterina

Table S1 presents the distribution of continuous variables for the overall sample and stratified by hospital. Demographic and epidemiological differences were observed between the two centers. At HUGJT, the screened population had a median age of 63 years, 54.6% were men, and 80.1% were born in Spain; while at HSC, the median age was 59 years, 53.8% were men, and 74.3% born in Spain (*P*_age_ = 0.01; *P*_born_ < 0.01). Compared with HUGJT, patients at HSC showed a higher proportion of individuals born in Africa (8.9% vs 3.7% for North Africa and 5.6% vs 1.6% for Sub-Saharan Africa) and Asia (1.3% vs 0.4% for East Asia), and a slightly lower proportion of individuals born in Europe (1.3% vs 2.6% for Eastern Europe and 0.6% vs 1.4% for Western Europe) (*P* < 0.01). The distributions of sex, age, and continental region of birth across the two hospitals are shown in Fig. 2, while the frequency of patients’ country of birth is presented in Table 1.

**Table 1:** Absolute frequency of recruited patients by country of birth

Country of birth	Absolut frequency
Spain	1583
Morocco	88
Honduras	37
Romania	23
Colombia	20
Gambia	19
Ukraine	11
France	10
Brazil	8
Ecuador	8
India	8
Argentina	7
Senegal	7
Ghana	6
Russia	6
Venezuela	6
Dominican Republic	5
Germany	4
Netherlands	4
Peru	4
Belgium	3
Bolivia	3
Bulgaria	3
Mali	3
Algeria	2
Cuba	2
Equatorial Guinea	2
Nicaragua	2
Nigeria	2
Paraguay	2
Philippines	2
Switzerland	2
United Kingdom	2
Angola	1
Armenia	1
Cameroon	1
Czech Republic	1
El Salvador	1
Greece	1
Guinea	1
Lebanon	1
Lithuania	1
Mauritania	1
Moldova	1
Poland	1
Serbia	1
Togo	1
Uruguay	1
United States of America	1

Regarding substance use, no differences were observed between Hospitals (*P* > 0.10) (Table S1). The proportions of active users and former users in the entire sample were as follows: tobacco (21.3% and 23.9%), alcohol (12.3% and 8.3%), cannabis (3.1% and 1.5%), cocaine (1.8% and 1.9%), heroine (0.3% and 0.4%), gamma hydroxybutyrate (GHB) (0.2% and 0.6%), ketamine (0.1% and 0.3%), and methamphetamine (0.3% and 0.4%) (Table S1).

With respect to previous infectious diagnoses, differences between Hospitals were observed for patients that had been previously diagnosed with a sexually transmitted disease (STD), being 3.1% in HUGJT and 6.3% in HSC (*P* < 0.01). No differences were observed for previously observed hepatitis infection (*P* > 0.05); being 1.1% with hepatitis C (ever HCV positive), and 1.1% with hepatitis B (ever HBV positive), in the whole sample (Table S1).

### HCV testing

The distribution of variables for the overall sample and stratified by hepatitis testing results are reported in Table S2 for HUGJT and in Table S3 for HSC. Anti-HCV testing was positive in 37 cases for HUGJT (2.2%) and 2 cases for HSC (0.7%), while follow-up HCV RNA testing identified 9 individuals (0.5%) for HUGJT and one participant (0.3%) for HSC with active infection, corresponding to 25.6% of anti-HCV positive (24.3% for HUGJT and 50% for HSC). Among individuals with active infection, 5 of them (50.0%) had not been previously diagnosed. Regarding linkage to care, 7 were successfully linked to specialist care; of these, 5 received treatment, one did not attend the second appointment, while another declined therapy. The remaining 3 patients died due to severe underlying conditions.

**Fig. 3:**
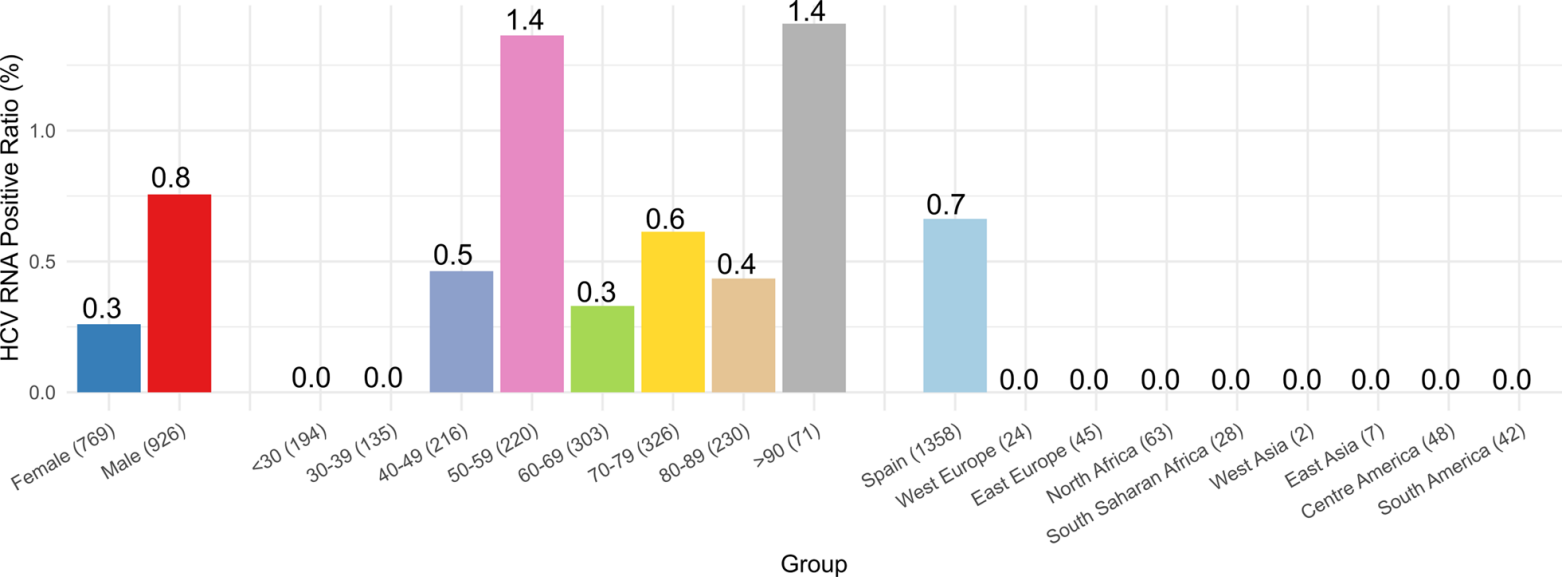
Rates of active HCV RNA positive testing among demographic groups

**Fig. 4:**
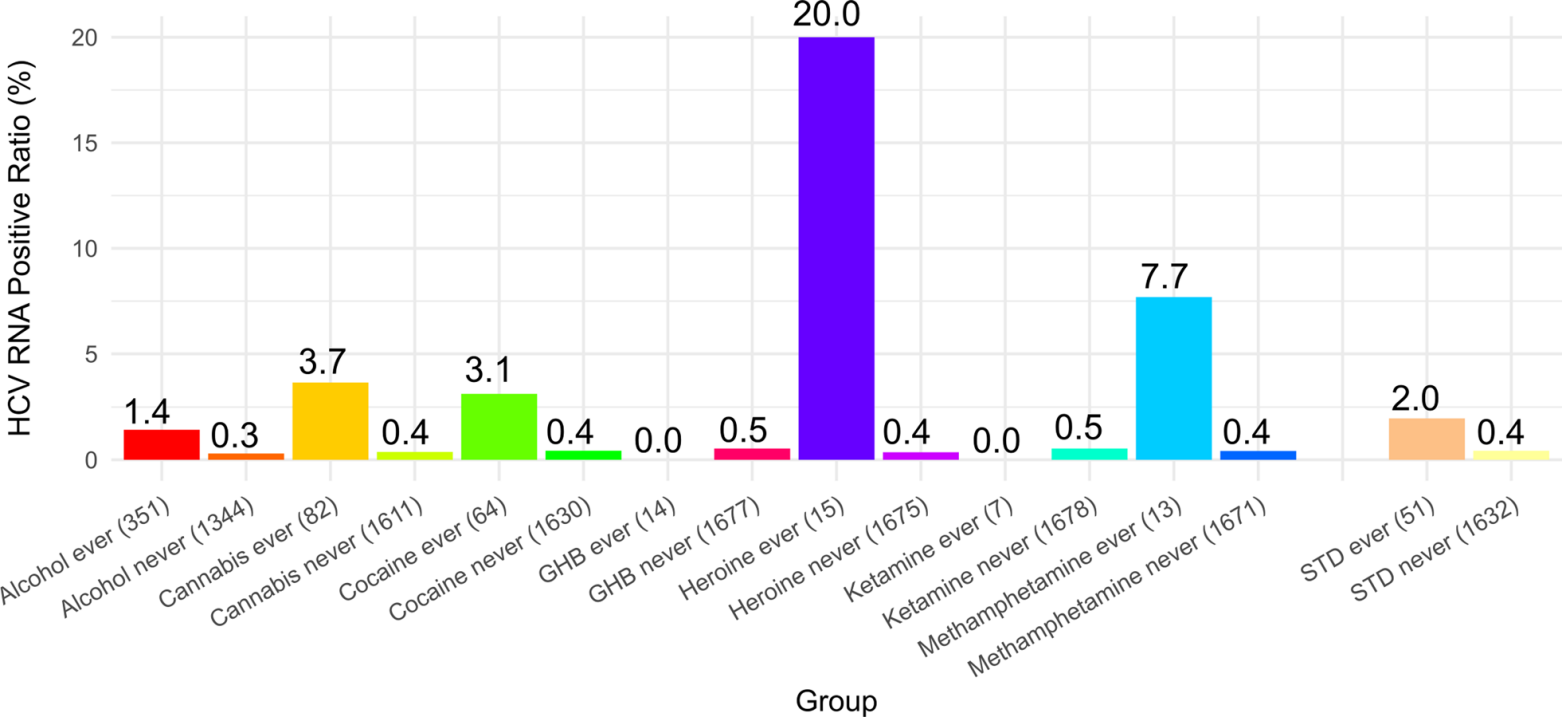
Rates of active HCV RNA positive testing among substance use and sexually transmitted diseases groups. GHB: Gamma hydroxybutyrate; STD: sexually transmitted diseases

Associated risk profiles were evaluated only for HUGJT (Table S2, Figs. 3 and 4). The analysis did not show statistically significant differences in active HCV infection according to sex, age group, or country of birth, although higher proportions were observed among men (0.8% vs 0.3% in women), individuals aged 50–60 years and those older than 90 years (1.4% in both cases), and patients born in Spain (0.7% vs 0.0% among those born abroad) (Fig. 3). Regarding substance use, a significantly higher prevalence of active HCV infection was observed among patients with a history of heroin use (20.0% vs 0.4%, *P* < 0.01) and among those reporting cannabis use (3.7% vs 0.8%, *P* < 0.01) (Fig. 4). No statistically significant differences were observed among patients with a history of sexually transmitted diseases (STD) (2.0% vs 0.4%) (Fig. 4).

### HBV testing

Regarding hepatitis B screening, a total of 11 individuals (0.6%) for HUGJT and 4 cases (1.3%) for HSC tested positive for HBsAg (Table S2 and Table S3), of whom 9 (60.0%) had no prior diagnosis. In terms of linkage to care, among those with a prior diagnosis, five individuals were already in active follow-up, while one patient had been lost to follow-up and was successfully re-linked to care through this screening. Among individuals without a prior diagnosis, four were successfully linked to specialist care, and one of them initiated treatment. Of the remaining five individuals, three did not attend their scheduled appointments, and two could not be linked to care because they were in transit.

**Fig. 5:**
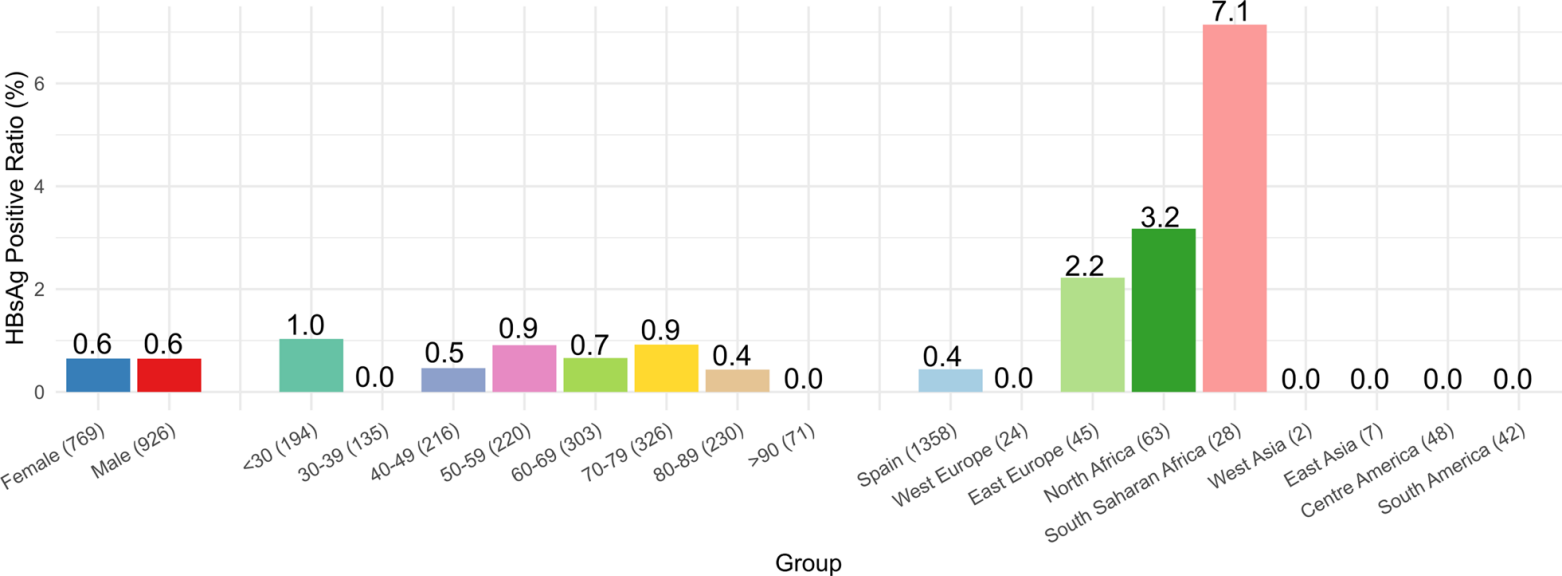
Rates of HBsAg positive testing among demographic groups

**Fig. 6:**
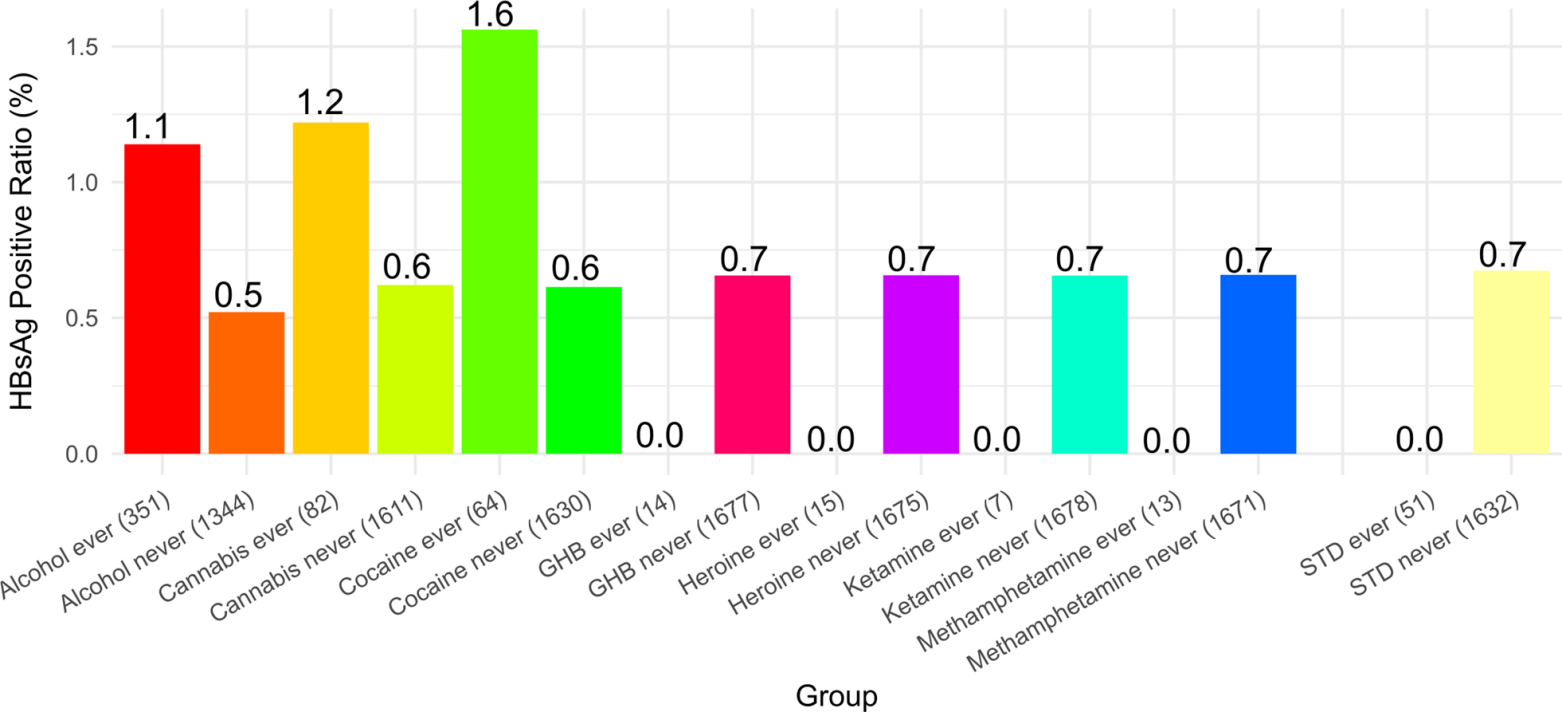
Rates of HBsAg positive testing among substance use and sexually transmitted diseases groups. GHB: Gamma hydroxybutyrate; STD: sexually transmitted diseases

Associated risk profiles were evaluated only for HUGJT (Table S2, Figs. 5 and 6). No statistically significant differences were observed according to sex or age group, although a higher proportion was noted among individuals younger than 30 years (1.0%). In contrast, significant differences were observed by place of birth, with higher prevalence among individuals born in East Europe (2.2%), North Africa (3.2%), and South-Saharan Africa (7.2%) (*P* < 0.01) (Table S2 and Fig. 5). No statistically significant differences were observed regarding substance use or among patients with a history of STD (Table S2 and Fig. 6).

## Discussion

This study evaluated the implementation of opportunistic HBV and HCV screening in the emergency departments (EDs) of two hospitals in the Girona Health Region and provides insights into implementation strategies, prevalence, risk profiles, and linkage-to-care outcomes in a real-world clinical setting.

The comparison between the two hospitals highlights the critical role that implementation strategies play in determining screening coverage. In the ED where screening was integrated into the EHR system and automatically performed unless the patient actively declined (opt-out approach), coverage reached 54.3%. In contrast, at the site where testing depended on an explicit physician request, coverage was markedly lower at 6.9%. These findings are consistent with studies from Europe and the United States showing that opt-out, EHR-integrated screening strategies can achieve uptake rates ranging from 50 to 90% [20], while also identifying cases that would likely be missed under conventional risk-based or physician-initiated testing protocols [26, 29, 30]. This difference underscores how removing procedural barriers and minimizing reliance on individual clinician initiative can substantially enhance screening reach. Overall, these results suggest that opt-out ED-based screening models can significantly improve both uptake and operational efficiency, supporting broader and more sustainable implementation of screening programs. Furthermore, non-targeted ED screening combined with effective linkage to care has been shown to outperform targeted approaches in identifying previously undiagnosed viral hepatitis infections [8], while also demonstrating cost-effectiveness. Beyond individual patient benefits, reducing the number of undiagnosed and untreated infections at the population level contributes to decreased transmission, and lower long-term healthcare costs [31, 32].

Due to slight demographic differences between the hospitals, consistent with official statistics from the Catalan Government, and the unequal contribution of each hospital to the overall sample, analyses were primarily focused on seroprevalence and associated risk profiles in the HUGJT cohort. Results from HSC were retained for comparison, but interpreted with caution given the smaller sample size and potential for biased estimates. The prevalence of active HCV infection observed in this study (0.5%) is consistent with recent estimates reported from ED-based screening programs in the Iberian Peninsula, which range between 0.17% and 0.73% for HCV RNA positivity [5, 14, 15, 33, 34]. The highest estimates were observed in the Hospital Vall d’Hebron (0.70–0.73%) [14, 34] in Barcelona, whereas lower estimates were reported in the Hospital de Bellvitge (0.33%) [33] and the Hospital Clínic (0.19%) [5]. Although these figures exceed national estimates from the Ministry of Health (0.12–0.22%), they are consistent with results from hospital-based screening strategies that focus on populations with potentially higher exposure risk [35]. In comparison with ED-based studies across Europe, the prevalence observed here falls within the lower end of the reported range (0.2–3.9%) [20]. In contrast, the prevalence of HBsAg positivity in this cohort (0.6%) is higher than previously reported national estimates (approximately 0.22%) and also exceeds values described in some Spanish ED-based studies, such as that conducted at Hospital Vall d’Hebron (0.5%) [14]. When compared with European ED-based data, this estimate lies at the upper end of the reported range (0.2–0.9%) [20]. The detection of previously unknown infection in 50% and 60% of the patients with active HCV and with HBV infection, respectively, further highlights the value of opportunistic screening in ED settings. These findings reinforce previous evidence suggesting that ED-based screening may capture individuals who remain undiagnosed through standard healthcare circuits, thereby contributing to improved case finding within elimination strategies [11].

The higher prevalence observed in this study, especially for HBV, may reflect the demographic characteristics of the ED population, which includes individuals with more complex health conditions, higher mobility, and potentially lower access to preventive healthcare services. The analysis of demographic and epidemiological characteristics revealed distinct patterns for HCV and HBV infection. Active HCV infection was more frequently observed among men (0.8%), individuals aged 50–59 (1.4%), and those born in Spain, although these associations were not statistically significant. These trends are consistent with the historical epidemiology of HCV infection in Spain, where transmission was largely associated with past healthcare exposures and injection drug use during the late twentieth century [36]. The strong association between HCV infection and a history of heroin use observed in this study (20.0%) supports the well-established link between injection drug use and HCV transmission. Although the prevalence associated with cannabis use should be interpreted cautiously due to the limited sample size and potential confounding factors, it may reflect overlapping behavioral risk patterns within substance-using populations.

In contrast, HBV infection showed a markedly different epidemiological pattern, with significantly higher prevalence among individuals born outside Spain, particularly those originating from South-Saharan Africa (7.1%), North Africa (3.2%), and Eastern Europe (2.2%). This is consistent with global HBV epidemiology and previous studies conducted in Spain and other European countries. These regions have higher endemicity levels for HBV, and migrants from such areas often acquire infection perinatally or during early childhood before migration [22, 37–39]. The findings therefore underscore the importance of considering country of origin when designing screening strategies and reinforce the need for targeted testing approaches among migrant populations, such as community-based or ED-based screening.

From a public health perspective, these findings support the role of EDs as an important entry point for opt-out viral hepatitis screening, particularly in regions with diverse populations and significant migrant communities. The ED setting provides an opportunity to reach individuals who may not otherwise access preventive services or routine primary care, such as migrant communities [24, 25]. Integrating systematic screening into ED workflows, particularly when combined with streamlined referral pathways and coordinated follow-up systems, may therefore contribute meaningfully to regional hepatitis elimination strategies and reaching the WHO elimination targets by 2030 [3].

This study has several limitations that should be considered when interpreting the results. First, differences in screening implementation between hospitals may have influenced patient selection and coverage rates; therefore, the two hospitals are not directly comparable. Second, screening was restricted to patients requiring blood tests for clinical reasons, which may introduce selection bias and limit generalizability to the broader ED population. Third, the number of confirmed active infections was relatively small, which may have limited the statistical power to detect associations in subgroup analyses. Additionally, the cross-sectional design for diagnoses limits the ability to establish causal relationships between risk factors and infection. Despite these limitations, the study provides valuable real-world data on the feasibility and outcomes of ED-based hepatitis screening in a regional healthcare context.

## Conclusions

Opt-out ED-based opportunistic screening for HBV and HCV proved effective in identifying previously undiagnosed infections (> 50% of active infections), particularly among populations with recognized epidemiological risk factors. The findings highlight the importance of an opt-out ED-based screening as a complementary strategy to existing public health programs and support the expansion of systematic, automated testing models. Strengthening LTC pathways and maintaining targeted approaches for high-risk populations, including migrants from high-endemic regions and people with a history of substance use, will be essential to maximize the contribution of ED screening to hepatitis elimination efforts.

## Supplementary Information

Additional file 1

## Data Availability

Clinical data included in this study is stored in a RedCAP project of the research centre account (Insititut d’Investigació Biomèdica de Girona Dr Josep Trueta – IDIBGI), and is managed in accordance with institutional confidentiality standards.

## Abbreviations

HBV: Hepatitis B virus
HCV: Hepatitis C virus
ED: Emergency departments
LTC: Linkage-to-care
WHO: World Health Organization
RSG: Regió Sanitària de Girona
PWID: People who inject drugs
HUGJT: Hospital Universitari de Girona Dr. Josep Trueta
HSC: Hospital Santa Caterina
HBsAg: Hepatitis B surface antigen
LCTG -ICS: Laboratori Clínic Territorial de Girona-Institut Català de la Salut
ECDC: European Centre for Disease Prevention and Control
DAAs: Direct-acting antivirals
EASL: European Association for the Study of the Liver
IDIBGI: Insititut d’Investigació Biomèdica de Girona Dr Josep Trueta
CEIM: Clinical Research Ethics Committee on Medicinal Products
GHB: Gamma hydroxybutyrate
STD: Sexually transmitted disease
EHR: Electronic health record
